# Assessing PARP trapping dynamics in ovarian cancer using a CRISPR-engineered FRET biosensor

**DOI:** 10.1016/j.crmeth.2025.101270

**Published:** 2025-12-30

**Authors:** Daniel Marks, Edwin Garcia, Sunil Kumar, Katie Tyson, Caroline Koch, Aleksandar P. Ivanov, Joshua B. Edel, Hasan B. Mirza, William Flanagan, Christopher Dunsby, Paul M.W. French, Iain A. McNeish

**Affiliations:** 1 Ovarian Cancer Action Research Centre, Department of Surgery and Cancer, Imperial College London, London W12 0NN, UK; 2 Francis Crick Institute, London NW1 1AT, UK; 3 Department of Physics, Imperial College London, London SW7 2AZ, UK; 4 Department of Chemistry, Imperial College London, London W12 0BZ, UK

**Keywords:** FLIM-FRET imaging, PARP1, PARP inhibitor, PARP trapping, DNA damage, ovarian cancer

## Abstract

Poly(ADP-ribose) polymerase inhibitors (PARPi) have revolutionized the treatment of ovarian high-grade serous carcinoma (HGSC), particularly in homologous recombination-deficient tumors. However, the emergence of resistance poses a critical challenge, as over 50% of patients relapse within 3 years. The mechanisms underlying changes in PARP trapping, a central aspect of PARPi efficacy, are not well understood, as current experimental methodologies lack resolution and throughput. To address this, we develop an intramolecular fluorescence resonance energy transfer (FRET)-based biosensor by CRISPR-Cas9 dual labeling of endogenous PARP1 with EGFP and mCherryFP in OVCAR4 cells. This biosensor enables real-time, single-cell analysis of PARP trapping dynamics. Using fluorescence lifetime imaging microscopy (FLIM), we reveal dose-dependent PARP trapping, differentiate the trapping efficiencies of four clinically approved PARPi, and observe reduced trapping in PARPi-resistant models *in vitro* and *in vivo*. This biosensor provides critical insights into PARPi resistance mechanisms, with implications for developing more effective therapies and advancing personalized treatment for ovarian cancer patients.

## Highlights

• Endogenous dual-labeling of PARP1 with FRET donor and acceptor via CRISPR-Cas9
• Fluorescence lifetime imaging enables real-time analysis of PARP1 trapping dynamics
• Biosensor distinguishes trapping efficiency among clinically approved PARP inhibitors
• Reduced PARP1 trapping correlates with acquired PARP inhibitor resistance

## Motivation

PARP inhibitors have transformed ovarian cancer treatment. However, resistance frequently arises, and the molecular mechanisms driving altered PARP1-DNA trapping remain poorly understood. Existing methods to measure PARP trapping lack the temporal/spatial resolution and throughput needed to capture heterogeneity. Here, we address these limitations by developing an intramolecular FRET biosensor that enables real-time, single-cell measurements of PARP trapping via high-content fluorescence lifetime imaging microscopy. This approach allows quantitative analysis of drug responses in live cells, enabling the study of how altered PARP1 trapping contributes to therapy resistance and guiding the development of more effective treatments.

## Graphical abstract

**Figure:**
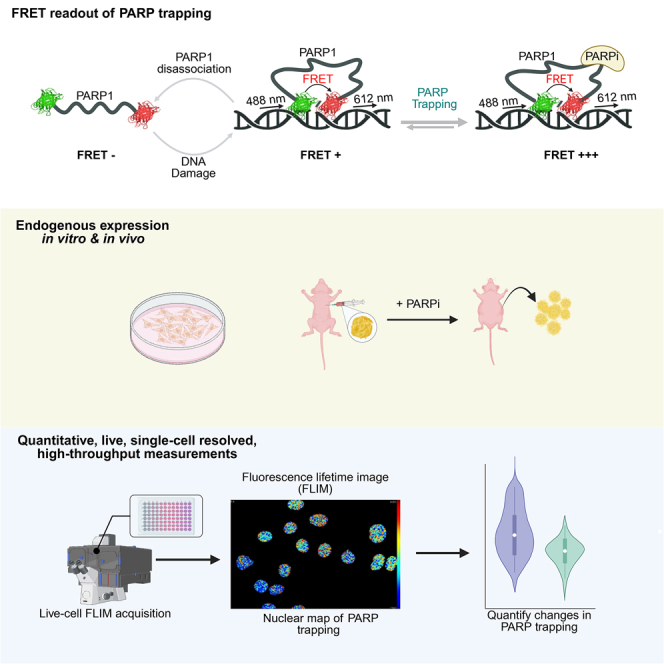

## Teaser

Marks et al. develop a CRISPR-engineered intramolecular FRET biosensor that enables high-content, single-cell quantification of PARP1 trapping via fluorescence lifetime imaging microscopy. This live-cell platform reveals how reduced PARP1 trapping may contribute to PARP inhibitor resistance, advancing dynamic analyses of DNA repair responses in ovarian cancer.

## Introduction

High-grade serous carcinoma (HGSC) is the most common and lethal subtype of ovarian cancer. Poly(ADP-ribose) polymerase inhibitors (PARPi) have transformed the management of *BRCA1/2*-mutated and homologous recombination (HR)-defective HGSC^1^^,^^2^ and also have activity in HR-proficient disease.^3^^,^^4^ However, most patients relapse within 3 years of treatment initiation, highlighting the need to understand and overcome PARPi resistance.

Upon the formation of single-strand DNA breaks, PARP1 attaches to damage lesions within seconds.^5^ NAD^+^ is used as a substrate to form PAR chain modifications (auto-PARylation), leading to chromatin remodeling and the recruitment of repair factors.^6^ PARPi function by preventing auto-PARylation, causing PARP to become trapped on DNA, which increases the steric complexity of lesion sites, resulting in the formation of double-strand DNA breaks (DSBs) during replication.^7^ When HR is functional, these DSBs can be repaired accurately. However, in cells lacking functional HR, these lead to genomic instability and cell death over multiple cell cycles.^8^^,^^9^

Multiple mechanisms of PARPi resistance have been identified in preclinical models, although few have been validated in clinical settings.^10^ Reactivation of functional HR, often due to *BRCA1/2* reversion mutations, is a key resistance mechanism.^11^ Other proposed mechanisms include increased drug efflux (MDR1 dependent),^12^ downregulation of poly(ADP-ribose) glycohydrolase (PARG),^13^ loss of 53BP1 expression,^14^^,^^15^ and diminished PARP trapping due to mutations in *PARP1*.^16^

Current methodologies to assess PARP trapping include chromatin immunoprecipitation (ChIP),^7^ which requires fixed, pooled cell populations, limiting its applicability to dynamic analyses. While fluorescence recovery after photobleaching partially overcomes this by enabling measurements in live cells, it is restricted to small populations (<100 cells), lacks the throughput needed to capture heterogeneity in response, and typically relies on overexpression of fluorescently tagged PARP1, which can alter protein kinetics.^17^^,^^18^

Here, we address these limitations by developing an intramolecular fluorescence resonance energy transfer (FRET) biosensor that enables real-time, single-cell resolved measurements of PARP trapping via high-content fluorescence lifetime imaging microscopy (FLIM). We demonstrate the utility of this biosensor by showing reduced PARP trapping in both *in vitro* and *in vivo* models of PARPi resistance. These findings pave the way for a deeper understanding of resistance mechanisms and inform the development of more effective and durable therapies for ovarian cancer patients.

## Results

### Endogenous dual labeling of PARP1 using CRISPR-Cas9

**Figure 1:**
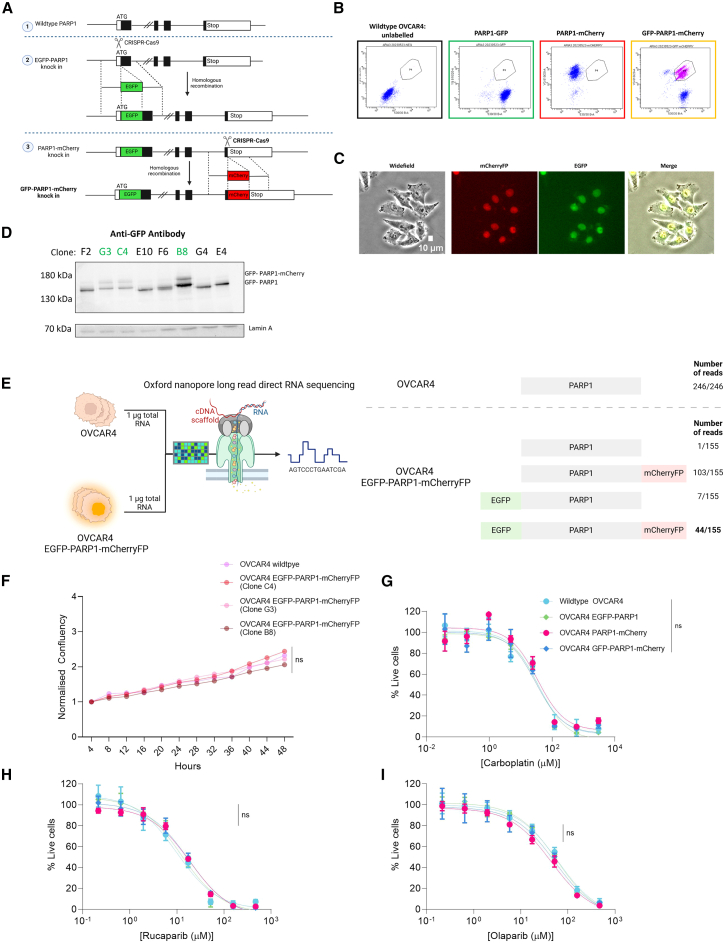
Endogenous dual labeling of PARP1 with donor (EGFP) and acceptor (mCherryFP) pair (A) Schematic of the sequential gene editing approach to knock in EGFP at the N terminus of PARP1, followed by mCherry at the C terminus. (B) Dual-labeled OVCAR4 cells were isolated by FACS. (C) Representative images of dual-labeled OVCAR4 EGFP-PARP1-mCherryFP cells showing phase, mCherryFP, EGFP, and merged images, scale bars: 10 μm. (D) Immunoblots were performed to assess labeling of PARP1; clones highlighted in green (G3, E4, and B8) showed dual-labeled EGFP-PARP1-mCherryFP. (E) Long-read direct RNA sequencing was achieved using Oxford nanopore sequencing of RNA extracted from parental OVCAR4 wild-type (WT) cells and EGFP-PARP1-mCherryFP cells. Isoform frequencies for each population are shown below. (F) Growth rate of each clone was assessed by acquiring time-lapse images over 48 h using the Incucyte S3 system at 10× magnification. Segmentation and confluency analysis performed in Incucyte software. Data were normalized to the confluency at t = 0 for each subsequent time point. (G–I) Sulforhodamine B (SRB) assays for OVCAR4 WT compared to the 3 CRISPR-generated cell lines treated with olaparib (G), carboplatin (H), or rucaparib (I). OVCAR4 WT (blue), OVCAR4 EGFP-PARP1 (green), OVCAR4 PARP1-mCherryFP (red), OVCAR4 EGFP-PARP1-mCherryFP (orange). Values were normalized to untreated controls to calculate live-cell values per treatment. One-way ANOVA was performed with multiple comparisons comparing the viability of each line to the parental cell line; all were non-significantly different, *n* = 3.

To generate a dual-labeled PARP1 FRET biosensor, a sequential CRISPR knockin (KI) strategy was employed, targeting the N termini and C termini of the *PARP1* locus, based on prior studies^19^ (Figure 1A). The initial CRISPR KI utilized a system comprising two plasmids (pDonor^PARP1.TS^ and SpCas9^D10A^:gRNA^PARP1^) for in *trans* paired nicking to insert EGFP at the N terminus of PARP1.^20^ Following transfection of OVCAR4 cells with these plasmids, fluorescence-activated cell sorting (FACS) was used to isolate EGFP-positive cells, yielding a homogenous OVCAR4 EGFP-PARP1-labeled population.

A second round of CRISPR KI targeted the C terminus of *PARP1* to insert mCherryFP. A rhodopsin-targeting vector (TV)^21^ was modified via restriction digestion and Gibson assembly, replacing the original EGFP sequence with mCherryFP and adapting the homology arms for the *PARP1* locus. The modified TV was co-transfected with gRNA enabling C-terminal PARP1-mCherryFP labeling. FACS was again performed to isolate cells co-expressing EGFP and mCherryFP (Figures 1B and 1C). From the heterogeneous dual-labeled population, single-cell clones were isolated (Figure S1A), and western blot analyses confirmed (Figure 1D) that three clones expressed both EGFP and mCherryFP. Clone B8 (used throughout) had ∼28.4% dual-labeled GFP-PARP1-mCherryFP fusion (Figure S1B).

To confirm KI, long-read direct RNA sequencing was performed using the MinION platform (Oxford Nanopore Technologies) using RNA extracted from parental OVCAR4 and OVCAR4 EGFP-PARP1-mCherryFP cells (clone B8) (Figure S1C). This showed a single PARP1 isoform in the wild-type population (246/246 reads) (Figure 1E). In the CRISPR-edited cells, PARP1-mCherryFP was identified in 103/155 reads, EGFP-PARP1 in 7/155 reads, and EGFP-PARP1-mCherryFP in 44/155 reads. This suggests that the dual-labeled PARP1 constitutes at least ∼30% of the total *PARP1* transcript pool. Dual labeling altered neither morphology nor cellular growth rates (Figures 1C–1F), nor sensitivity to carboplatin (Figure 1G) or PARPi rucaparib and olaparib (Figures 1H and 1I), validating the use of these models for subsequent studies.

### FLIM-FRET analysis validates dual-labeled OVCAR4 cells as a biosensor for PARP trapping

**Figure 2:**
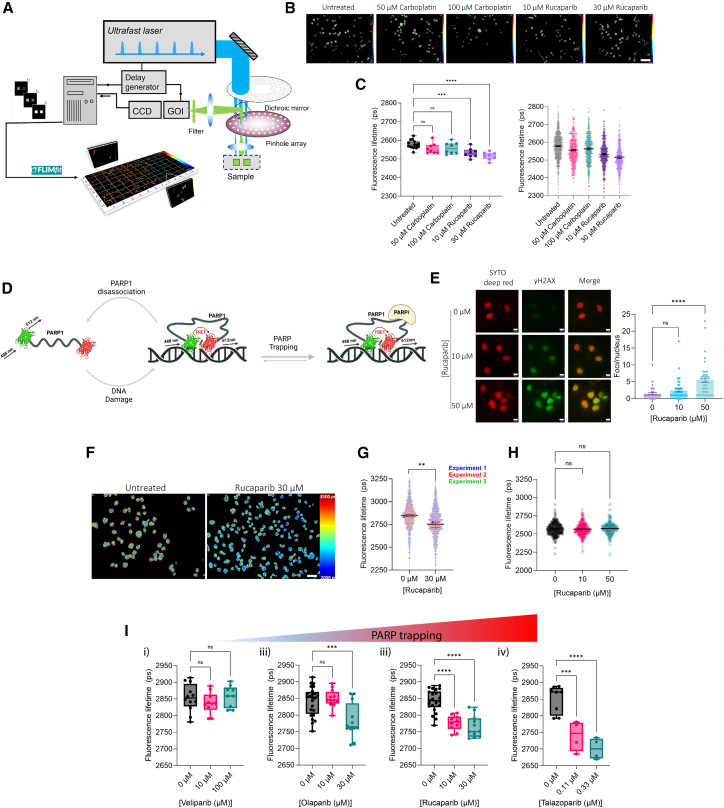
EGFP-PARP1- FRET biosensor detects changes in PARP trapping, rather than DNA damage (A) System diagram of wide-field time-gated fluorescence lifetime imaging microscope. Intensity images are acquired at various time gates, and fluorescence decays are fitted using global fitting of a double-exponential decay in FLIMfit. (B) Representative FLIM images per condition from HCA FLIM-FRET assay, images are pseudo-colored by mean fluorescence lifetime. (C) Fluorescence lifetime of FRET biosensor donor upon treatment with carboplatin (50 and 100 μM) or rucaparib (10 and 30 μM) for 1 h, compared to untreated controls. Values are summarized as FOV averages (left) or single-cell mean fluorescence lifetimes (right). One-way ANOVA (Kruskal-Wallis) with Dunn’s multiple comparison test was used to assess statistical significance between treatment conditions ∗∗∗*p* < 0.0001 and ∗∗∗∗*p* < 0.0001. (D) Schematic of the FRET biosensor under PARP-trapping conditions. (E) Representative images of γH2AX foci per cell following 1 h of rucaparib exposure at 0, 10, or 50 μM in OVCAR4 EGFP-PARP1-mCherryFP cells, quantification shown with mean and SEM values. Scale bars: 10 μm, *n* = 3 biological replicates. (F and G) Representative images of segmented OVCAR4 EGFP-PARP1-mCherryFP nuclei, pseudo-colored by mean fluorescence lifetime values with untreated (left) and 30 μM rucaparib-treated values for 1 h shown in (G). (H) Single-cell mean fluorescence lifetime values are plotted per condition and color-coded by the experimental repeat they are part of. Fluorescence lifetime of EGFP in donor-only controls (OVCAR4 EGFP-PARP1) exposed to 0, 10, or 50 μM rucaparib for 1 h. Statistical significance between treatment conditions was assessed using an unpaired *t* test: ns, *p* > 0.05, ∗∗*p* < 0.01, *n* = 3 biological repeats. (I) Change in FRET biosensor donor fluorescence lifetime in OVCAR4 EGFP-PARP1-mCherryFP-expressing live cells, upon treatment for 1 h with differing PARPi, veliparib (i), olaparib (ii), rucaparib (iii), or talazoparib (iv) at various doses. One-way ANOVA (Kruskal-Wallis) with Dunn’s multiple comparison test was used to assess statistical significance between treatment conditions.

To evaluate the efficacy of dual-labeled OVCAR4 EGFP-PARP1-mCherryFP cells as a FRET biosensor, wide-field time-gated FLIM high-content analysis (HCA) was performed (Figure 2A). Fluorescence lifetime images of EGFP (the FRET donor) were globally fitted using FLIMfit software.^22^ Regions of interest corresponding to individual nuclei were segmented to enable single-cell quantification and population-level analysis under different treatment conditions. An initial experiment assessed the response to changes in DNA damage and PARP trapping following treatment with carboplatin and rucaparib, respectively (Figures 2B and 2C). Changes in fluorescence lifetime were analyzed at both the field-of-view (FOV) and single-cell levels, revealing consistent trends. Carboplatin treatment induced non-significant reductions in mean fluorescence lifetime compared to untreated cells. This was not unexpected, as platinum drugs induce DNA crosslinking, leading to DSBs, which are not the target of PARP1-mediated repair. In contrast, rucaparib caused significant dose-dependent reductions in fluorescence lifetime, with mean values of 2,532 and 2,516 ps at 10 and 30 μM rucaparib, respectively (*p* < 0.05). This suggests that the FRET biosensor specifically reports changes in PARP trapping rather than general DNA damage. We hypothesize that this specificity arises from a higher proportion of biosensor molecules persisting in a high FRET efficiency state (Figure 2D). Notably, FOV- and single-cell analyses were highly consistent, with only a 0.07% average variation, confirming the reliability of population-level fluorescence lifetime measurements. To confirm the specificity of the signal, FLIM assays were conducted using three distinct clonal populations of OVCAR4 biosensor-expressing cells (Figure S1D). All clones exhibited a dose-dependent reduction in fluorescence lifetime in response to rucaparib exposure. Clone B8, which demonstrated the largest dynamic range in lifetime change, was selected for subsequent experiments. γH2AX phosphorylation immunofluorescence showed a dose-dependent increase in γH2AX foci per cell, confirming that 1 h exposure to PARPi was sufficient to induce DNA damage (Figure 2E).

Separate repeat measurements carried out at weekly intervals corroborated the reproducibility of changes in PARP trapping upon rucaparib treatment (Figures 2F and 2G). Statistically significant reductions in fluorescence lifetime were consistently observed in biosensor-expressing cells treated with 30 μM rucaparib for 1 h compared to untreated controls. Conversely, OVCAR4 EGFP-PARP1 (donor only) cells did not exhibit significant fluorescence lifetime changes under identical conditions, underscoring the specificity of the biosensor (Figure 2H).

To evaluate the biosensor’s capacity to differentiate the trapping potency of different PARPi, cells were treated with veliparib, olaparib, rucaparib, or talazoparib. Distinct fluorescence lifetime profiles were observed, consistent with published rankings of PARP trapping efficiency (Figure 2I). Veliparib resulted in no significant difference in mean lifetime values (2,854, 2,838, and 2,858 ps for 0, 10, and 100 μM, respectively), indicating minimal PARP trapping. Olaparib and rucaparib both induced dose-dependent reductions in fluorescence lifetime (olaparib: 2,842, 2,850, and 2,782 ps for 0, 10, and 30 μM, respectively; rucaparib: 2,842, 2,777, and 2,766 ps for 0, 10, and 30 μM, respectively). The reductions were statistically significant at 30 (olaparib) and ≥10 μM (rucaparib). Talazoparib exhibited the most potent PARP-trapping effects, with significant reductions in fluorescence lifetime detected at 0.11 μM (2,850, 2,740, and 2,701 ps for 0, 0.11, and 0.33 μM, respectively; *p* < 0.01). These findings validate that dual-labeled PARP1 FRET can discern PARP trapping in live cells with high sensitivity and reproducibility. The rank order of PARPi trapping potency observed here, talazoparib > rucaparib ≈ olaparib > veliparib, closely aligns with prior literature,^7^ further supporting the biosensor’s utility for investigating PARP trapping in live cells.

### FLIM-FRET biosensor detects PARP trapping in isogenic HR-proficient and HRD cells

**Figure 3:**
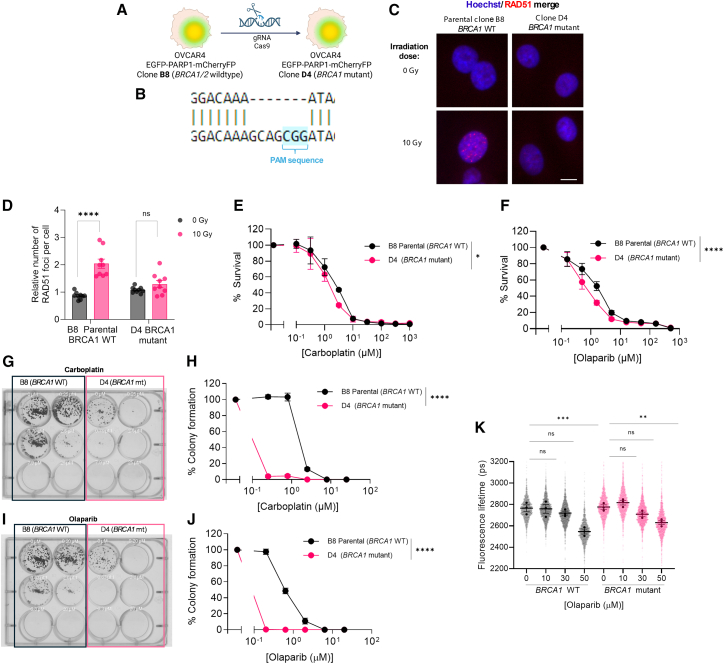
Measuring PARP trapping in isogenic HR-proficient and HRD models generated by CRISPR-mediated BRCA1 deletion (A) Schematic representation of CRISPR-Cas9-mediated generation of *BRCA1*-mutant clones from parental OVCAR4 EGFP-PARP1-mCherryFP cells. Clone B8 (*BRCA1* WT) serves as the isogenic control while clone D4 harbors the *BRCA1* mutation. (B) Sequence alignment generated using EMBOSS Water showing the 7 bp deletion at the PAM sequence (blue) of exon 7 gRNA target in clone D4 compared to WT sequence in parental clone B8. (C) Representative immunofluorescence images showing RAD51 foci formation (red) in Hoechst-stained nuclei (blue) following 0 or 10 Gy irradiation in *BRCA1* WT (B8) and *BRCA1*-mutant (D4) cells. Scale bars, 10 μm. (D) Quantification of RAD51 foci per cell. Each point represents the mean value of an FOV, bars show mean ± SEM. Statistical significance was assessed using one-way ANOVA with Tukey’s multiple comparison test, ∗∗∗∗*p* < 0.0001, ns, not significant, *n* = 3 biological replicates with >1,000 cells analyzed per condition. (E and F) Cell viability curves assessed by SRB assay following 6 days treatment with carboplatin (E), or olaparib (F) in *BRCA1* WT (B8) and BRCA1-mutant (D4) cells. Survival scores are normalized to vehicle-treated controls. Error bars represent SEM, *n* = 3 biological replicates. Statistical significance between genotypes was assessed by nonlinear regression and extra sum-of-squares F test, ∗*p* < 0.05, ∗∗∗∗*p* < 0.0001. (G–J) Representative crystal-violet-stained clonogenic assay plates and quantification of total stained area (percent colony formation) following 10 days of treatment with carboplatin (G and H) or olaparib (I and J) in *BRCA1* WT (B8) and *BRCA1*-mutant (D4) cells. Data are expressed as percent colony formation relative to vehicle-treated controls. Error bars represent mean ± SEM; *n* = 3 technical replicates. Statistical comparisons were performed using two-way ANOVA with Bonferroni correction for multiple comparison test (∗∗∗∗*p* < 0.0001). (K) Live-cell FLIM-FRET analysis of PARP trapping in *BRCA1* WT and mutant cells treated with increasing doses of olaparib (0–50 μM) for 1 h. Violin plots show single-cell EGFP fluorescence lifetime distributions. One-way ANOVA (Kruskal-Wallis) with Dunn’s multiple comparison test was used to assess statistical significance between treatment conditions, ∗∗*p* < 0.01, ∗∗∗*p* < 0.001, ns, not significant, *n* = 3 biological replicates.

To establish a genetically defined homologous recombination-deficient (HRD) model, CRISPR-Cas9 editing was applied to OVCAR4 EGFP-PARP1-mCherryFP biosensor cells, targeting exon 7 of *BRCA1* (Figure 3A). Genomic cleavage detection assay confirmed successful CRISPR-Cas9 targeting with 23% modification efficiency (Figures S2A and S2B). Sequence alignment of the parental clone B8 (*BRCA1* wild type) with the derived clone D4 confirmed a 7 bp deletion at the gRNA target site adjacent to the PAM motif, consistent with frameshift disruption of BRCA1 (Figure 3B).

We first examined HR repair capacity by quantifying RAD51 nuclear foci formation after irradiation. In *BRCA1* wild-type cells, exposure to 10 Gy ionizing radiation induced a significant increase in RAD51 foci per nucleus relative to unirradiated controls (Figures 3C and 3D). In contrast, *BRCA1*-mutant D4 cells failed to mount a comparable response, with no significant change in foci number compared to untreated controls. This confirms that loss of BRCA1 function impairs RAD51 recruitment and HR activity.

We next assessed functional differences in drug sensitivity between isogenic wild-type and *BRCA1*-mutant OVCAR4 EGFP-PARP1-mCherryFP cells. Viability assays revealed that the *BRCA1*-deficient clone D4 displayed enhanced sensitivity to both carboplatin and olaparib compared to wild-type B8 cells (Figures 3E and 3F). In colony formation assays, *BRCA1*-deficient cells exhibited at least a 25-fold reduction in carboplatin tolerance (Figures 3G and 3H) and a ≥229-fold reduction in olaparib tolerance (Figures 3I and 3J) relative to wild-type controls, consistent with the expected synthetic lethality of HRD cells to DNA crosslinking agents and PARP inhibition.

To investigate PARP trapping dynamics, we performed live-cell FLIM-FRET imaging using our biosensor in both wild-type and *BRCA1*-mutant cells treated with olaparib. In both genotypes, olaparib treatment resulted in significant dose-dependent decreases in donor fluorescence lifetime, indicating PARP trapping (Figure 3K).

In BRCA1 wild-type cells, the mean fluorescence lifetime decreased from 2,765 (untreated) to 2,547 ps (*p* < 0.001) following 50 μM olaparib treatment. Similarly, BRCA1-mutant cells showed a reduction from 2,776 to 2,630 ps under the same conditions (*p* < 0.01). Importantly, untreated cells from both genotypes displayed comparable baseline fluorescence lifetimes (2,765 vs. 2,776 ps, *p* > 0.999), demonstrating that the observed differences in PARP trapping are specifically induced by olaparib treatment rather than inherent genotypic differences.

Together, these findings validate the successful generation of isogenic *BRCA1*-mutant PARP-trapping biosensor cells. The BRCA1-deficient clone demonstrates classical hallmarks of HRD: increased platinum/PARPi sensitivity and defective RAD51 focus formation. These characteristics support its utility as a genetically defined model for investigating PARP1 dynamics in HRD contexts.

### Decreased sensitivity to PARPi correlates with lower PARP trapping *in vitro*

**Figure 4:**
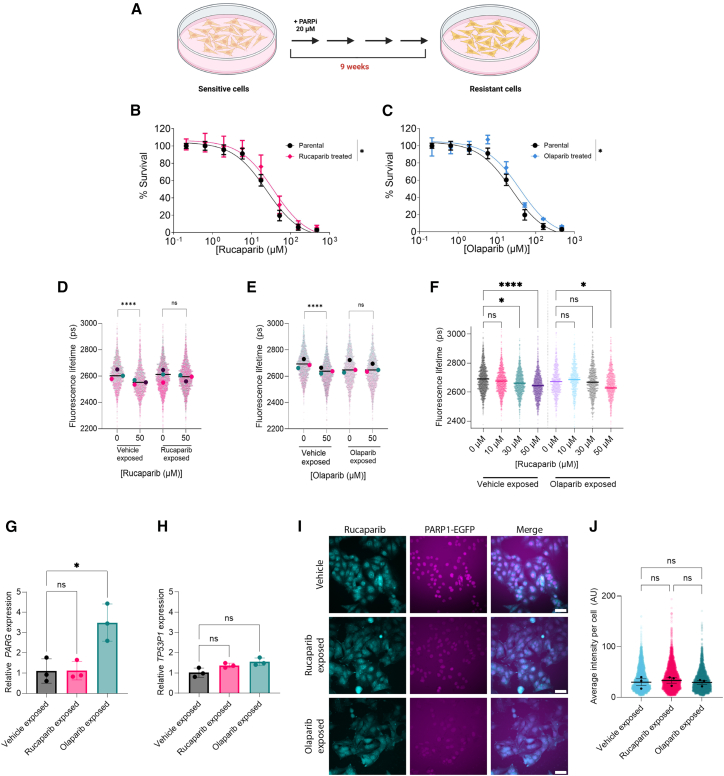
Induction of resistance *in vitro* induces a reduction in PARP trapping as assessed by the EGFP-PARP1-mCherryFP FRET biosensor (A–C) (A) Schematic of the treatment protocol to induce *in vitro* resistance to olaparib or rucaparib. OVCAR4 EGFP-PARP1-mCherryFP cells were treated daily with 20 μM olaparib or rucaparib for 8 weeks. Resistance was assessed by SRB assay to (B) rucaparib or (C) olaparib and compared to vehicle-treated parental OVCAR4 EGFP-PARP1-mCherryFP cells. *t* tests were performed to confirm IC_50_ values, *n* = 3 biological repeats. (D and E) (D) Live-cell FLIM-FRET HCA assays were performed to assess changes in donor lifetime of the EGFP-PARP1-mCherryFP FRET biosensor upon 1 h treatment with rucaparib or (E) olaparib. Single-cell FLIM data show population-level distributions of mean-weighted fluorescence lifetime. Data are shown as mean ± SEM, *n* = 3 biological repeats, and statistical significance was assessed using one-way ANOVA. (F) Change in FRET biosensor donor fluorescence lifetime of vehicle-exposed (left) or olaparib-exposed (right) cells upon treatment with rucaparib at 0, 10, 30, or 50 μM for 1 h. One-way ANOVA (Kruskal-Wallis) with Dunn’s multiple comparison test was used to assess statistical significance between treatment conditions. (G and H) (G) RT-qPCR analysis of PARG and (H) TP53BP1 relative mRNA expression normalized to GAPDH expression. *n* = 3 biological repeats. (I) Representative images of rucaparib and EGFP fluorescence in OVCAR4 EGFP-PARP1-mCherryFP cells following vehicle, rucaparib, or olaparib exposure *in vitro* for 9 weeks. (J) Quantification of average intracellular rucaparib intensity of images in (I), each point is a single cell average and error bars represent population median. *n* = 3 biological repeats.

To induce resistance *in vitro*, OVCAR4 EGFP-PARP1-mCherryFP cells were treated daily with 20 μM olaparib or rucaparib (doses approximating their 72 h IC_50_ values) for 9 weeks, with daily medium replenishment (Figure 4A). Populations derived from this protocol are termed “olaparib-exposed” and “rucaparib-exposed” in subsequent analyses. The sensitivity of these cells to their respective PARPi (Figures 4B and 4C) decreased significantly, with an increase in IC_50_ values from 22 to 36 μM (rucaparib) and from 23 to 39 μM (olaparib), confirming that the long-term treatment protocol effectively induced PARPi resistance. Both rucaparib- and olaparib-exposed cells also demonstrated reduced sensitivity to cisplatin (Figure S3); the cisplatin IC_50_ in rucaparib-exposed cells was 17 μM, and 13 μM in olaparib-exposed cells, compared to 5 μM for parental cells.

To assess changes in PARP trapping, FLIM-FRET HCA assays were performed on PARPi-exposed cells. Following re-treatment with rucaparib, there was no significant change in fluorescence lifetime in the rucaparib-exposed population (2,594–2,589 ps, *p* = 0.9822), in contrast to a significant reduction in parental cells (2,616–2,553 ps, *p* < 0.0001) (Figure 4D). A similar trend was observed in the olaparib-exposed population, where re-exposure to olaparib produced no significant change in fluorescence lifetime (2,665–2,656 ps, *p* < 0.6836) while the parental population underwent a significant reduction in lifetime (2,700–2,664 ps, *p* < 0.0001) (Figure 4E). To evaluate cross-resistance, olaparib-exposed cells were treated with rucaparib, which produced no statistically significant reduction in fluorescence lifetime at 50 μM (2,672 and 2,629 ps for 0 and 50 μM, respectively; *p* = 0.111). As observed previously, fluorescence lifetimes were significantly reduced in parental cells at 30 μM (2,690–2,644 ps; *p* < 0.0001) (Figure 4F). Taken together, these results suggest that a reduction in PARP trapping may be a common mechanism driving resistance to PARPi.

Expression of *ABCB1* (*MDR1*), *PARG*, and *TP53BP1* was assessed using quantitative reverse-transcription PCR (RT-qPCR) (Figures 4G and 4H). *ABCB1* was undetectable in both PARPi-exposed populations. No significant decrease in *PARG* expression was observed, although a significant increase was observed in the olaparib-exposed cells (*p* = 0.009); *TP53BP1* expression also remained unchanged.

To assess further whether reduced PARP trapping resulted from lower intracellular drug concentrations, we utilized the intrinsic fluorescence of rucaparib and quantified fluorescence per cell as a proxy for intracellular concentration (Figure S4). There were no significant differences in median population fluorescence values between sensitive and PARPi-exposed populations (*p* = 0.9058, *p* = 0.9973) following treatment at 10 μM rucaparib (Figures 4I and 4J).

### PARP trapping is decreased following *in vivo* exposure to olaparib

**Figure 5:**
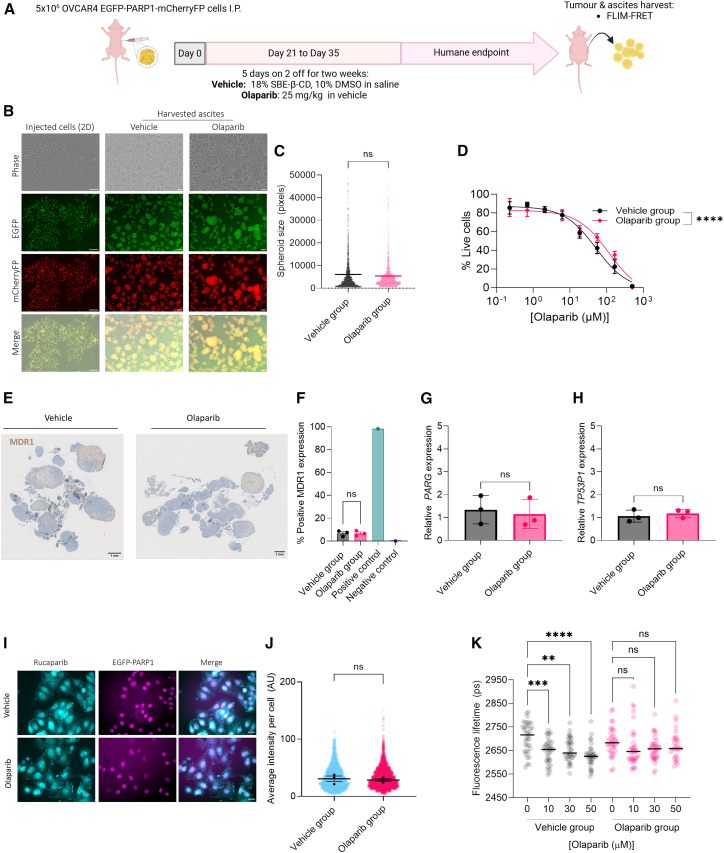
*In vivo* resistance generation to olaparib leads to a reduction in PARP trapping (A) OVCAR4 EGFP-PARP1-mCherryFP cells were injected intraperitoneally (I.P.) into female CD1 nude mice and allowed to grow for 21 days before mice were treated with vehicle or olaparib (5 mg/kg) daily for 14 days. Mice were culled upon reaching humane endpoint, and omental tumors and ascites were harvested. (B) Representative images showing OVCAR4 EGFP-PARP1-mCherryFP cells grown in 2D monolayers before injecting into CD1 nude mice (left) and the ascites harvested from the peritoneal cavity of these mice after 90 or 92 days for vehicle- and olaparib-treated groups, respectively. Images were acquired using the Incucyte S3 with 10× objective. (C) Quantification of average spheroid area per condition. (D) Viability assays were performed on the ascites cells grown in 2D, treated with olaparib for 72 h. Survival scores are normalized to vehicle-treated controls. Error bars represent SEM, *n* = 3 biological replicates. (E) Representative IHC images of MDR1 (brown stain) from OVCAR4 EGFP-PARP1-mCherryFP tumors, scale bars: 1 mm. (F) OVCAR4 EGFP-PARP1-mCherryFP omental tumors harvested at day 90 (vehicle) or 92 (olaparib) were stained for MDR1 by IHC. The number of MDR1-positive tumor cells was quantified using QuPath.^23^ Statistical significance was tested using an unpaired *t* test. (G and H) (G) RT-qPCR analysis of PARG and (H) TP53BP1 relative mRNA expression normalized to GAPDH expression. *n* = 3 biological repeats. (I) Representative images of rucaparib intrinsic fluorescence (left), EGFP-PARP1 (middle), and merged image (right) in the vehicle- or olaparib-exposed populations, scale bars: 25 μm. (J) Quantification of median rucaparib signal per cell. *n* = 3 biological repeats. (K) Fluorescence lifetimes of EGFP measured in FLIM assays of OVCAR4 EGFP-PARP1-mCherryFP cells that had been harvested from mice ascites of each group. Cells retrieved from vehicle- and olaparib-treated mice were incubated with olaparib (0–50 μM) for 1 hour prior to fluorescence microscopy. Error bars show mean and SEM value, *n* = 3 biological replicates. One-way ANOVA (Kruskal-Wallis) with Dunn’s multiple comparison test was used to assess statistical significance between treatment conditions.

To investigate the potential changes in PARP trapping *in vivo*, nude mice bearing intraperitoneal OVCAR4 GFP-PARP1-mCherryFP xenografts were treated with olaparib (25 mg/kg) or vehicle for 2 weeks on a 5-days-on/2-days-off schedule (Figure 5A). Thereafter, mice were monitored until reaching humane endpoint, at which point tumor cells were isolated from ascitic fluid or peritoneal lavage for subsequent monolayer culturing (Figure 5B). There was no difference in ascites spheroid size in the vehicle- and olaparib-treated groups, despite some differences in morphology (Figure 5C).

Viability assays revealed a significant increase in resistance to olaparib in the olaparib-treated group compared to the vehicle-treated group (*p* = 0.0002) (Figure 5D). Expression of MDR1 in OVCAR4 EGFP-PARP1-mCherryFP tumor sections was assessed using immunohistochemistry (IHC) (Figure S5), showing no significant change between the vehicle- (H-score range, 4–9) and olaparib (H-score range, 5–9)-treated groups (*p* = 0.804) (Figures 5E and 5F). We also assessed the expression of *ABCB1*, *TP53BP1*, and *PARG* using RT-qPCR (Figures 5G and 5H). *ABCB1* was again undetectable. Both *TP53BP1* and *PARG* remained unchanged between the vehicle- and olaparib-exposed groups.

Another possible explanation for observing reduced PARP trapping could be due to reduced intracellular drug concentration due to extracellular matrix (ECM) remodeling or modulation of influx or non-MFR1 efflux pumps. To test this, we again assessed the fluorescence intensity of rucaparib per cell as a readout of intracellular concentration (Figures 4I and 4J) and again found no differences in intracellular concentration, suggesting that this is not a mechanism altering PARP trapping in these cells.

Finally, HCA FLIM-FRET assays were performed to evaluate PARP trapping (Figure 5K). In vehicle-treated cells, fluorescence lifetimes significantly decreased upon olaparib exposure in a dose-dependent manner, from 2,716 ps in untreated cells to 2,654 (*p* = *0.0008)*, 2,639 (*p* = 0.0032), and 2625 ps (*p* = < 0.0001), at 10, 30, and 50 μM, respectively, confirming that dose-dependent PARP trapping remained even after *in vivo* selection and *ex vivo* culturing. By contrast, cells from the olaparib-treated group showed no significant changes in fluorescence lifetime upon olaparib treatment (from 2,683 ps in untreated cells to 2,646 [*p* = 0.6983], 2,657 [*p* = 0.3497], and 2,658 ps [*p* = 0.9903] for 10, 30, and 50 μM, respectively). These findings demonstrate that *in vivo* exposure to olaparib not only decreases sensitivity to the drug but also results in reduced levels of PARP trapping.

## Discussion

Here, we describe the development of an endogenous PARP1 FRET-based biosensor that enables high-resolution, real-time measurement of PARP trapping in live cells. By combining this biosensor with FLIM readouts, we achieve robust, single-cell quantitative analysis of PARP1 conformational dynamics, a proxy for PARP trapping. This approach can facilitate high-content screening to investigate mechanisms of PARPi resistance. Using this system, we identified reduced PARP trapping in populations with decreased PARPi sensitivity, providing key preclinical evidence linking altered PARP dynamics to resistance. This innovation represents a significant advance, offering new opportunities to study PARPi resistance in clinically relevant settings.

The lack of effective methodologies for studying PARP trapping has limited our understanding of its role in PARPi resistance. Our biosensor directly addresses this gap and can discern differences in trapping efficiency across clinically approved PARPi, validating findings from traditional population-based assays such as ChIP, while also offering dynamic and single-cell insights. These capabilities are essential for a fuller understanding of PARPi resistance.

Our findings build upon earlier work that described a fluorescent sensor for PARP1 conformational changes in purified proteins.^19^ We extended this method to live cells by employing sequential rounds of CRISPR-Cas9 KI for dual labeling of endogenous PARP1 with FRET pairs. Dual labeling of PARP1 was confirmed using long-read RNA sequencing, where approximately 30% of *PARP1* transcripts covered the full 5,386 nucleotide dual-labeled transcript. However, since nanopore sequencing reads unidirectionally (starting from the C terminus) and the median read length was 918 nucleotides with only 1.7% of reads over 5,000 nucleotides, we believe that the results are likely to be a significant underestimation of the true proportion of dual-labeled transcripts. This also explains the observed enrichment of PARP1-mCherryFP fragments in the long-read sequencing. Based on estimated PARP1 levels (0.2–1 × 10^6^ copies per cell),^24^^,^^25^ we estimate there will be least 60,000 copies of the PARP1 FRET biosensor per cell. Importantly, we showed clearly that the PARP1 sensor does not impede cellular fitness or PARP1 functionality. Cell size, morphology, growth rate, and sensitivity to PARPi and platinum therapy were all unaffected following CRISPR labeling to express the FRET sensor.

We utilized HCA FLIM-FRET assays in the HGSC cell line OVCAR4^26^ to monitor PARP trapping in thousands of live cells per condition with and without drug perturbation. We found that the sensor could recapitulate previously measured trends in PARP-trapping potency of multiple PARPi, including veliparib, olaparib, rucaparib, and talazoparib. Importantly, we also demonstrated that the biosensor effectively detects PARP trapping in both HR-proficient and HRD contexts. Both genotypes showed comparable baseline fluorescence lifetimes and significant, dose-dependent decreases upon olaparib treatment, confirming that the biosensor’s functionality is preserved regardless of HR status. This finding is particularly significant as it demonstrates that PARP trapping occurs in HRD cells, where it likely contributes to the synthetic lethality underlying their enhanced PARPi sensitivity.

This gave us confidence in the utility of the biosensor and facilitated our assessment of PARP trapping dynamics in disease models where the PARP-trapping status was unknown. This uncovered consistent reductions in PARP trapping in populations that had reduced sensitivity to PARPi following continuous exposure to a constant dose of PARPi for 9 weeks *in vitro*. Moreover, trapping was impaired in PARPi-resistant cells when exposed to both their original treatment and alternative PARPi, suggesting class-wide adaptations. Additionally, these cells showed decreased cisplatin sensitivity, indicating potential cross-resistance between PARPi and platinum-based therapies. These findings align with reports of shared resistance mechanisms between PARPi and platinum drugs^27^ and are consistent with clinical observations of reduced platinum efficacy post PARPi progression in the SOLO2 trial.^28^ Further studies are needed to elucidate the molecular pathways driving these resistance mechanisms and their broader implications.

Lastly, we generated biosensor-expressing tumors *in vivo* and measured PARP trapping in cells derived from vehicle- or olaparib-treated tumors *ex vivo*, uncovering reductions in PARP trapping in the treated populations. These results were in accordance with our *in vitro* observations, despite distinct treatment protocols.

Several avenues for future research could build upon this work. The biosensor-expressing cells could be grown in spheroids or co-cultures to assess PARP trapping dynamics in *in vitro* models that better recapitulate the tumor microenvironment. Efforts to streamline the CRISPR-Cas9-based dual-labeling process, such as employing a lentiviral system, could facilitate broader adoption of this methodology in varied models, including patient samples and *in vivo*, where experimental tools to measure PARP trapping are currently lacking. Additionally, the real-time capabilities of our biosensor enable longitudinal studies to examine the kinetics of PARP-trapping changes during resistance development, revealing whether reduced trapping precedes or follows other resistance mechanisms. The biosensor’s potential extends to benchmarking the trapping potency of future PARPi and assessing trapping efficiency in clinical samples, paving the way for more personalized therapeutic decisions. Additionally, this platform could support the development of PARPi that exclusively target PARP1, addressing recent findings implicating PARP2 trapping in toxicity.^29^ Ultimately, this biosensor offers a transformative tool for understanding and overcoming PARPi resistance, providing a foundation for future therapeutic strategies that improve clinical outcomes.

### Limitations of the study

Despite its strengths, this study has several limitations. First, our protocols produced only modest IC_50_ increases in resistance models, in contrast with larger c.10-fold increases observed in prior work where resistance was induced over 11 months using increasing doses of olaparib over time.^30^ Thus, it is possible that additional mechanisms, beyond altered PARP trapping, are driven by treatment protocols that increase dosage over time. However, our protocols were chosen to recreate clinical schedules, where patients receive PARPi at fixed daily doses without any dose escalation. Additionally, while we successfully generated isogenic *BRCA1*-mutant biosensor cells to study PARP trapping in HRD contexts, our models cannot capture all clinically relevant resistance mechanisms. Notably, we cannot model *BRCA* reversion mutations, which are identified in approximately 25% of *BRCA*-mutated HGSC progressing on PARPi treatment.^11^^,^^31^ A significant proportion of resistance in these patients remains unexplained, and unanswered questions remain regarding whether reduced PARP trapping is a direct driver of resistance in *BRCA*-mutant tumors or other cells with intrinsically higher levels of genomic instability. Nonetheless, the dynamics of PARP trapping versus BRCA reversion in resistance development warrant further exploration.

## Resource availability

### Lead contact

Requests for further information and resources should be directed to and will be fulfilled by the lead contact, Professor Iain A. McNeish (i.mcneish@imperial.ac.uk).

### Materials availability

Plasmids and cell lines generated in this study are available from the lead contact upon request.

### Data and code availability

• Raw nanopore sequencing data are available via ENA under accession number ENA: PRJEB87876 and are publicly available as of the date of publication. Microscopy data reported in this paper will be shared by the lead contact upon request.
• Python pyDNA code is available at https://doi.org/10.5281/zenodo.17570791.
• Any additional information required to reanalyze the data reported in this paper is available from the lead contact upon request.

## Acknowledgments

We acknowledge funding from 10.13039/501100000289Cancer Research UK (A29368) and the 10.13039/100010438Francis Crick Institute, which receives its core funding from 10.13039/501100000289Cancer Research UK (FC001999), the 10.13039/501100000265Medical Research Council (FC001999), and the 10.13039/100010269Wellcome Trust (FC001999). D.M. acknowledges a PhD studentship funded by 10.13039/501100000289Cancer Research UK (A30808), and W.F. acknowledges a PhD studentship funded by the Physics Department at Imperial College London. I.A.M. is an NIHR Senior Investigator, and C.K. holds an EPSRC Doctoral Prize Fellowship. This was also supported by the Imperial 10.13039/501100000272NIHR Biomedical Research Center (Surgery and Cancer theme), but no 10.13039/501100000272NIHR funding was used to support *in vivo* experiments. The funders had no role in study design, data collection and analysis, decision to publish, or preparation of the manuscript.

The authors acknowledge the technical expertise of Martin Kehoe in establishing the FLIM microscopes. FACS was performed with the aid of the LMS NIHR Flow Cytometry Facility, Imperial College London. Rhodopsin-targeting vector (EGFP) was kindly gifted by Professor Izumi Oinuma of the Laboratory of Cell and Molecular Biology, University of Hyogo, Japan. AW69_pDonor^PARP1.TS^ and AM70_pgRNA^PARP1^ plasmids were generated and kindly gifted by Dr. Manual A.F.V. Gonçalves, Department of Cell and Chemical Biology, Leiden University Medical Center, the Netherlands. Yuriy Alexandrov of the Physics Department at Imperial College London and the Francis Crick Institute assisted with the implementation and application of the FLIM data analysis tools. Some figures were created in BioRender, license reference D.M. (2025) https://BioRender.com/x54zecp.

## Author contributions

Conceptualization, I.A.M., P.M.W.F., and C.D.; data acquisition, D.M., E.G., S.K., K.T., C.K., and W.F.; data analysis, D.M., E.G., S.K., C.K., and H.B.M.; software and code, D.M., E.G., S.K., and H.B.M.; supervision, A.P.I., J.B.E., P.M.W.F., C.D., and I.A.M.; paper writing, D.M., I.A.M., C.D., and P.M.W.F. All authors reviewed and approved the paper.

## Declaration of interests

I.A.M. has acted on advisory boards for AstraZeneca, GSK, and pharma& and declares prior institutional grant support from AstraZeneca.

## STAR★Methods

### Key resources table

**Table:** 

REAGENT or RESOURCE	SOURCE	IDENTIFIER
**Antibodies**		

Anti-PARP1 (mouse)	Cell Signaling	Cat# 9542S; RRID: AB_2160739
Anti-GFP (mouse)	Merck Millipore	Cat# 11814460001; RRID: AB_390913
Anti-Laminin A/C (mouse)	Cell Signaling	Cat# 2032; RRID: AB_2617138
Secondary anti-mouse	DAKO	Cat# P0447; RRID: AB_2617737
Secondary anti-rabbit	DAKO	Cat# P0446; RRID: AB_2617138
MDR1/ABCB1 (E1Y7S)	Cell Signaling	Cat# 13978; RRID: AB_2798537
γH2AX-AF488	Cell Signaling	Cat# 20304; RRID: AB_2798841
Anti-RAD51 (rabbit)	Abcam	Cat# EPR4030(3); RRID: AB_2722613
Nano-Secondary anti-rabbit, Alexa Fluor 647	ChromoTek	Cat# srbAF647-1; RRID: AB2827587

**Bacterial strains**		

JM109 competent cells	Promega	Cat# P9751
High competency *StbI3*	NEB	Cat# B90205
One Shot™ TOP10 Chemically Competent *E. coli*	Invitrogen	Cat# K4575J10

**Chemicals, peptides, and recombinant proteins**		

Rucaparib	APExBIO	Cat# A8893
Olaparib	Selleckchem	Cat# S1060
Carboplatin	Enzo Life Sciences	Cat# ALX-400-041-M025
Talazoparib	Selleckchem	Cat# S7048
Veliparib	Selleckchem	Cat# S1004

**Critical commercial assays**		

RNeasy Micro Kit	Qiagen	Cat# 74104
High-Capacity cDNA Reverse Transcription Kit	Applied Biosystems	Cat# 4368814
Direct RNA Sequencing Kit	Oxford Nanopore Technologies	Cat# SQK-RNA004
TOPO™ TA Cloning™ Kit for Sequencing	Invitrogen	Cat# K4575J10
GeneArt™ Genomic Cleavage Detection Kit	Invitrogen	Cat# A24372

**Deposited data**		

Raw sequencing data	This paper	ENA: PRJEB87876

**Experimental models: Cell lines**		

Human: OVCAR4	ATCC	SCC258
Human: OVCAR4 EGFP-PARP1	This paper	N/A
Human: OVCAR4 PARP1-mCherry	This paper	N/A
Human: OVCAR4 EGFP-PARP1-mCherry	This paper	N/A
Human: OVCAR4 EGFP-PARP1-mCherry *BRCA1* mt (Clone D4)	This paper	N/A

**Experimental models: Organisms/strains**		

Mouse: Female CD1-nude (Crl:CD1-Foxn1^nu^)	Charles River, U.K.	Crl:CD1-Foxn1^nu^

**Oligonucleotides**		

Oligonucleotides used in the paper	This paper	See Table S1.

**Recombinant DNA**		

mCherry-H2B	Addgene	#20972
pX330-U6-chimeric-BB-Cbh-hSpCas9	Addgene	#42230
pST1374-N-NLS-flag-linker-Cas9-D10A	Addgene	#51130
pgRNA^PAPR1^	Chen et al.^20^	N/A
pDonor^PARP1^	Chen et al.^20^	N/A
Rhodopsin EGFP targeting vector backbone	Matsuda et al.^21^	N/A

**Software and algorithms**		

ImageJ	Schindelin et al.^32^	https://imagej.net/
*FLIMfit*	Warren et al.^22^	https://flimfit.readthedocs.io/en/latest/
GraphPad Prism	N/A	https://www.graphpad.com/
ApE plasmid editor	Davis et al.^33^	https://jorgensen.biology.utah.edu/wayned/ape/
pyDNA	Pereira et al.^34^	https://github.com/pydna-group/pydna
Epi2Me, “wf-transcriptomes”	Epi2Me Labs	https://github.com/epi2me-labs/wf-transcriptomes
EMBOSS water	EMBL	http://emboss.open-bio.org/
CHOPCHOP	Labun et al.^35^	https://chopchop.cbu.uib.no/
Python pyDNA code	This paper	https://doi.org/10.5281/zenodo.17570791

### Experimental model and study participant details

#### Mice

*In vivo* experiments were performed at the Central Biological Services facility, Imperial College London and approved by the Imperial College Animal Welfare & Ethics Review Body (AWERB). Experiments were performed under the authority of project license numbers PA780D61A and PP1321516. All experiments conformed to UK Home Office regulations under the Animals (Scientific Procedures) Act 1986, including Amendment Regulations 2012. Female CD1-nude (Crl:CD1-*Foxn1*^*nu*^) mice (Charles River, U.K.) between 6 and 7 weeks old were exclusively used. Only female mice were used as this study investigates ovarian cancer, which does not occur in males.

#### Cell cultures and analyses

OVCAR4 cells were obtained from ATCC. Cell identity was validated by 16 locus STR validation. Cells were tested for mycoplasma infection regularly using MycoAlert detection kit.

### Method details

#### Cell culture

OVCAR4 cells were cultured in DMEM, supplemented with 10% heat-inactivated fetal bovine serum, 2 mM L-Glutamine and 100 μg/mL penicillin/streptomycin. All cells were grown in 5% CO_2_, 37°C with humidity, and used for a maximum of 10 passages.

#### CRISPR/Cas9 knock-in of EGFP and mCherryFP at endogenous PARP1 locus

Open-access Python package pyDNA was used for in silico plasmid sequence design.^34^ CHOPCHOP (https://chopchop.cbu.uib.no) was used to design guide RNA (gRNA) targeted to the N- or C-termini of *PARP1*. 5′ and 3′ homology arms (HA) to *PARP1* were synthesized by PCR.

For C-terminal labeling, the mCherry fragment was amplified from a mCherry-H2B plasmid (Addgene 20972) with complementary overhangs to the 5′ and 3′ HA. The targeting vector backbone (kindly gifted by Matsuda and Oinuma^21^) was digested with AscI and SwaI, isolating the 2004 bp backbone fragment. The 5′ and 3′ homology arms (sequences taken from Perez-Leal et al.^36^) were amplified via PCR, using primers designed to create fragments with complementary overhangs ∼22 bp in length. Gibson assembly reactions were performed at 50°C for 1 h using the NEBuilder HiFi DNA assembly cloning kit (NEB E5520S), combining DNA fragments with overlapping sequences at equal molar ratios with the digested backbone sequence. The reaction product was transformed in NEB 5α competent *E. coli* (NEB C2987) and isolated using QIAprep Spin Miniprep Kit (Qiagen 27104). This PARP1 C-terminal targeting vector was used with the same gRNA previously used by Perez-Leal et al., GTCAATTTTAAGACCTCCCTG, which was ligated into pX330-U6-chimeric-BB-Cbh-hSpCas9 plasmid (Addgene 42230).^36^

For N-terminal knock-in (KI), *in trans* paired nicking was used. Two plasmids kindly gifted by Chen et al.^20^ (pgRNA^PAPR1^, pDonor^PARP1^) were subsequently transfected withpsT1374-N-NLS-flag-linker-Cas9-D10A (Addgene 51130). These three plasmids constituted the CRISPR system for N-terminal targeting of PARP1.

For CRISPR transfections, 2 × 10^5^ OVCAR4 cells were plated overnight in 6-well plates in antibiotic-free medium and transfected with 1 μg of each plasmid using Viafect (Promega E4981). Fluorescence positive cells were isolated after 24–48h using FACS, and single cell colonies were expanded.

#### BRCA1 deletion in OVCAR4 EGFP-PARP1-mCherryFP cells

TrueGuide synthetic gRNA targeting BRCA1 exon 11 (Invitrogen) was reconstituted to 100 μM in nuclease-free water. 2 × 10ˆ5 OVCAR4 EGFP-PARP1-mCherryFP cells were plated overnight in 6-well plates. Cells were transfected with 7.5 pmol sgRNA and 7.5 pmol TrueCut Cas9 protein v2 (Invitrogen, A36496) complexed with Lipofectamine CRISPRMAX (Invitrogen, CMAX00008) in Opti-MEM (Gibco 31985-070) according to manufacturer’s protocols. After 24h, transfection medium was replaced with complete medium. Indel frequency was determined 48h post-transfection using the GeneArt Genomic Cleavage Detection Kit (Thermo Fisher Scientific, A24372) following manufacturer’s protocols. Clonal dilution was performed seeding 0.5 cells/well, clones were screened by RAD51 immunofluorescence assay, extracting DNA from candidate clones. Sanger sequencing was performed (Azenta Life Sciences) and pairwise alignment of sequences with the parental cells was performed using EMBOSS Water (EMBL-EBI).

#### Long-read direct RNA-sequencing

Direct RNA sequencing (DRS) was performed using the MinION platform (Oxford Nanopore Technologies, MIN-101B) and RNA flow cells (ONT, FLO-MIN004RA). Libraries were generated using 1 μg total RNA with the Direct RNA Sequencing Kit (Oxford Nanopore Technologies, SQK-RNA004) according to the manufacturer’s instructions. Base calling was performed with using Dorado. Basecalled sequencing data were processed using the wf-transcriptomes pipeline within the Epi2Me software. The workflow includes read preprocessing, alignment using minimap2, transcript assembly and quantification, quality control and validation. Reads were visualised using Integrative Genomics Viewer (IGV, www.igv.org/) and a ratio of reads per isoform of PARP1 was calculated per sample.

#### Automated wide-field time-gated FLIM microscope and FLIM data acquisition

The instrument used for these experiments is described in detail in.^37^ Quasi-confocal FLIM was implemented upon a motorized inverted epifluorescence microscope (Olympus IX-81, incorporating a ZDC autofocus) with a spinning disc scanner (Yokogawa, CSU-X) used for all experiments. Upon drug treatment, imaging 96-well plates were immediately transferred to the microscope chamber at 37°C, 5% CO_2_, mounted on a motorized x-y stage (Märzhäuser Wetzlar GmbH, SCAN IM 120 × 80) and left for 1 h for temperature equalisation before optically sectioned FLIM acquisitions. A 20× air objective lens (Olympus UPlanApo 20×) with an NA of 0.7 was used. For excitation of EGFP, a supercontinuum laser (NKT, FIU-15 PP) with a tuneable output selector (NKT, SuperK Varia) was used to provide pulsed excitation radiation at 488 ± 20nm at a repetition rate of 78 MHz.

FRET biosensor-expressing OVCAR4 cells (1 × 10^4^ per well) were seeded onto a fibronectin (Sigma F0895) coated (3 μg/cm^2^) 96-well glass imaging plate (Greiner 655892) in CO_2_-dependent imaging medium 48 h before imaging and maintained at 37°C with 5% CO_2_ during imaging. A 488/532 dichroic mirror (Semrock, Di01-T488/532-13 × 15 × 0.5) directed the excitation beam, with power adjusted to ∼200 μW at the sample plane. Fluorescence emission was collected through a 520/35 nm filter (Semrock, FF01-520/35-25) and detected using a gated optical intensifier (Kentech Instruments, HRI-HL) coupled to a cooled CCD camera (Photometrics Retiga R1) via a 0.7× demagnification relay. The gating signal was set to a 4 ns width and synchronized to the laser pulses, with time delays controlled by the μManager plugin openFLIM-HCA (https://github.com/imperial-photonics/openFLIM-HCA). FLIM images were acquired at seven time delays with a total FLIM data acquisition time of ∼10 s per field of view (FOV).

Data were saved as OME-TIFF files and analyzed using the open-source MATLAB software *FLIMfit*.^22^ The instrument response function (IRF) was accounted for in each experiment by measuring the fluorescence decay of a reference dye (75μM Coumarin 6 in ethanol) at 25ps intervals over a 12.5 ns pulse period. Additionally, quenched fluorescein (2.5μL of 250μM fluorescein in 100 μL 2M potassium iodide) was used as a secondary reference with a very short lifetime (<200ps) lifetime. The Coumarin 6 image dataset was fitted using reference reconvolution employing the spatially averaged quenched fluorescein decay as the reference decay. The fluorescence lifetime of the quenched fluorescein was input as 180ps as determined from a previous time-correlated single-photon counting measurement. The FLIMfit option “Create IRF Shift Map” was used to calculate the spatially varying IRF. This shift map was used subsequently when fitting FLIM data to correct for spatial variations in the IRF across the HRI FOV. Measurements were taken before and after each imaging session to confirm stability. A time-varying background was measured in well containing only cell culture media. Global double-exponential fitting was performed per field of view (FOV), and weighted mean lifetime values were extracted on a per-cell, per-condition basis.

#### Sulforhodamine B (SRB) cell growth assay

Cell lines were seeded at 7,500 cells per well in 96-well plates. The following day, medium was replaced with drug-containing medium or a vehicle-only control (DMSO). Unless otherwise stated in figure legends, cells were treated for 72 h. For the *BRCA1* CRISPR experiment, 1,000 cells per well were seeded, and treatment was extended to 6 days, with fresh drug-containing medium added every 3 days.

At the endpoint, cells were fixed with 50% (w/v) trichloroacetic acid (TCA) (Sigma-Aldrich T6399) for 1 h at 4°C, washed five times with water, and dried overnight at room temperature (RT). Cells were stained for 1 h at RT with 0.4% (w/v) sulforhodamine B (SRB; Sigma-Aldrich S1402) in 1% (v/v) acetic acid (Thermo Fisher 10005920). Plates were washed five times with 1% (v/v) acetic acid to remove unbound dye and air-dried overnight. Bound SRB dye was solubilized in 100 μL 10 mM Tris base (Sigma-Aldrich T1699) for 10 min on an orbital shaker, and absorbance was measured at 510 nm using a TECAN plate reader. Three technical replicates were included per condition. Medium-only wells were averaged and subtracted from all readings to remove background. Data were normalized to vehicle-control wells.

#### Clonogenic assay

Clonogenic assays were performed to assess long-term survival of OVCAR4 EGFP-PARP1-mCherryFP cells and their derived clones B8 (*BRCA1* wild-type) and D4 (*BRCA1* mutant) following drug treatment. Cells were seeded at 650 cells per well in 12-well plates and allowed to adhere for 48 h under standard tissue culture conditions. Cells were treated with either olaparib (maximum concentration 20 μM) or carboplatin (maximum concentration 25 μM) prepared as ½-log serial dilutions (five concentrations total) alongside a vehicle control (VC; equivalent final DMSO concentration). Treatments were maintained for 10 days, with fresh medium and drug replenished every 3 days.

At endpoint, cells were washed twice with PBS then fixed and stained with 0.1% (w/v) crystal violet (Sigma-Aldrich C6158) in 25% methanol for 15 min at room temperature. Plates were rinsed thoroughly with distilled water and air-dried. Plates were imaged using an ImageQuant 4000 (Cytiva) in transillumination mode with a 1 s exposure time. Image analysis was performed in FIJI (ImageJ, NIH). As individual colonies could not be resolved, total stained area per well was quantified following thresholding. The measured area for each treated well was normalised to the mean area of vehicle-treated controls (set as 100%) to yield percent colony formation.

#### Western blot

Protein samples were resolved by SDS-PAGE using a Bio-Rad Mini-PROTEAN Tetra System. SDS running buffer (20 mM Tris-HCl, 190 mM glycine, and 0.1% SDS in distilled H_2_O) was added to fill the electrophoresis tank, carefully ensuring no leaks between adjacent gels. Samples were loaded, and electrophoresis was conducted at 80 V for 30 min, followed by 140 V until the tracking dye reached the gel’s bottom.

Protein transfer to a nitrocellulose membrane was performed via wet transfer. Sponges, filter papers, and nitrocellulose membranes were pre-soaked in transfer buffer (20 mM Tris-HCl, 190 mM glycine, 20% methanol in distilled H_2_O). Protein transfer was performed at 200 mA for 90 min. Ponceau Red (Sigma-Aldrich, P7170) staining was used to confirm protein transfer on the nitrocellulose membrane, followed by three washes in TBS-T to remove excess stain.

Membranes were then blocked in 5% (w/v) BSA (Sigma-Aldrich, A3294) in TBS-T for 1 h at room temperature. Primary antibodies were prepared in BSA solution and incubated with the membranes overnight at 4°C on a tube roller. After incubation, unbound primary antibodies were removed with three 5-min washes in TBS-T. Membranes were then incubated with secondary antibodies, diluted in 5% (w/v) powdered milk (Millipore, 70166), for 1 h at room temperature on a tube roller. Excess secondary antibody was removed with three 5-min washes in TBS-T. Finally, membranes were developed using enhanced chemiluminescence (Cytiva Life Sciences, RPN2106) and imaged with the GE ImageQuant LAS 4000 Biomolecular Imager system.

#### γH2AX immunofluorescence

Cells were fixed in ice-cold methanol for 10 min, permeabilized in permeabilization solution (PS) (0.5% Triton X-100 (Amresco, 0694) in PBS) for 1h at RT. Blocking solution (BS) (PS + 10% FBS) was then added for 1 h at RT. γH2AX-AF488 (Cell Signaling Technology, 20304) staining was performed at 1:10,000 dilution in BS at 4°C, overnight. SYTO Deep Red (Thermo Fisher, S34900) staining was performed for 1h at room temperature (1:1000 in PBS). Three PBS washes were performed, and samples were subsequently imaged using a 100×, 1.4 NA objective Olympus UplanSApo objective lens with HILO illumination. AF488 was imaged with 462nm laser excitation and SYTO Deep Red was imaged with 635nm laser excitation.

#### RT-qPCR

Culture medium was aspirated from 6-well plates and 350 μL RLT buffer was added and frozen at −80°C. Plates were thawed on ice and 70% ethanol was added and transferred into a RNeasy Micro Kit column (Qiagen, 74104). RNA extraction was performed as per the manufacturer’s instructions, genomic DNA was digested using RNase-Free DNase (Qiagen, 79254). RNA was eluted in 30μL nuclease-free water, concentration and quality were estimated using Nanodrop and Qubit 3.0 analysis.

1 μg RNA was used in each 20 μL cDNA reaction with the High-Capacity cDNA Reverse Transcription Kit (Applied Biosystems, 4368814) with cycles of 25°C 10 min, 37°C 120 min, and 85°C 5 min. The resulting cDNA was diluted in 140μL nuclease-free water. qRT-PCR reaction was performed using 9 μL cDNA, 1 μL primer and 10 μL TaqMan Universal Master Mix II no UNG (Thermo Fisher Scientific, 4440040). TaqMan primer probes (*GAPDH* (Hs02786624_g1), *PARG (*Hs00608254_m1*), TP53BP1* (Hs00996827_m1), *ABCB1* (Hs00184500_m1), Thermo Fisher Scientific). Samples were loaded in a 96-well plate (Applied Biosystems, 4311971) and sealed with optical plate seal (Applied Biosystems, 4346907) and analyzed on a StepOnePlus (Applied Biosystems). Gene expression was normalised to *GAPDH.*

#### MDR1 IHC

MDR1/ABCB1 (E1Y7S) 1:300 Ab dilution (Cell Signaling, 13978). Negative control was a cytospin of parental PEO1 cells, positive control was a cytospin of PEO1 cells overexpressing MDR1.

#### Imaging intrinsic fluorescence of rucaparib

A custom-built multi-beam, multi-photon, multi-well plate microscope (M^3^M) built on the opensource *openFrame* microscope (Cairn Research, UK) was used. Prior to imaging, cells were treated for 1 h with 10 μM rucaparib. The sample was excited via widefield illumination using an LED light engine at 365 nm (CoolLED, pE-4000) coupled via a 360/50 emission filter and 460/50 emission filter (Chroma, 49000 ET) to image intracellular rucaparib using a 20×0.95NA water immersion objective (Nikon, MRD77200). Scripts were written to use CellPose Cyto3 model for automated whole cell segmentation in Fiji to define ROIs within which the rucaparib total intensity was quantified to assess single cell intracellular drug concentration.

#### *In vivo* experiments

OVCAR4 GFP-PARP1-mCherryFP cells were injected intraperitoneally (I.P.) (5 × 10^6^ cells in 200 μL PBS) into CD1-nude (Crl:CD1-*Foxn1*^*nu*^) mice (Charles River, U.K.). On days 15–28 inclusive, 25 mg/kg olaparib (Selleckchem S1060) dissolved in 18% SBE-β-CD (MedChemExpress HY-17031), 10% DMSO in saline was injected I.P. Mice were culled when the first in each group reached humane endpoint and tumors harvested into PBS on ice. If there was no ascites present, peritoneal lavage (5 mL of 2 mM EDTA in PBS) was performed to isolate ascites spheroids, which were centrifuged at 200 x g for 5 min and resuspended in TrypLE Express Enzyme (Gibco 12604013) for 10 min, 37°C. Disassociated spheroids were subsequently resuspended in medium and cultured under standard conditions.

### Quantification and statistical analysis

Data are presented as arithmetic mean ± SD or median ±SD, as stated in figure legends. The exact values of n (biological replicates) are provided in figure legends. Statistical analyses were performed using GraphPad Prism v.9.4.1 and a *p*-value ≤0.05 was considered statistically significant. For FLIM-FRET assays each technical replicate typically contained thousands of cells per condition. No statistical methods were used to predetermine sample sizes.

## Supplemental information

Document S1. Figures S1–S5 and Table S1

Document S2. Article plus supplemental information
