# Supplementary material for: Assessing PARP trapping dynamics in ovarian cancer using a CRISPR-engineered FRET biosensor
Source: Cell Rep Methods. 2025 Dec 30;6(1):101270. doi: 10.1016/j.crmeth.2025.101270 (PMC12853179; doi:10.1016/j.crmeth.2025.101270)
Supplement: Document S1. Figures S1–S5 and Table S1 [file mmc1.pdf]

Cell Reports Methods, Volume *6*

Supplemental information

Assessing PARP trapping dynamics in ovarian cancer using a CRISPR-engineered FRET biosensor

Daniel Marks, Edwin Garcia, Sunil Kumar, Katie Tyson, Caroline Koch, Aleksandar P. Ivanov, Joshua B. Edel, Hasan B. Mirza, William Flanagan, Christopher Dunsby, Paul M.W. French, and Iain A. McNeish

Figure S1. Generation and characterisation of biosensor expressing clonal populations, related to Figure 1 and 2.

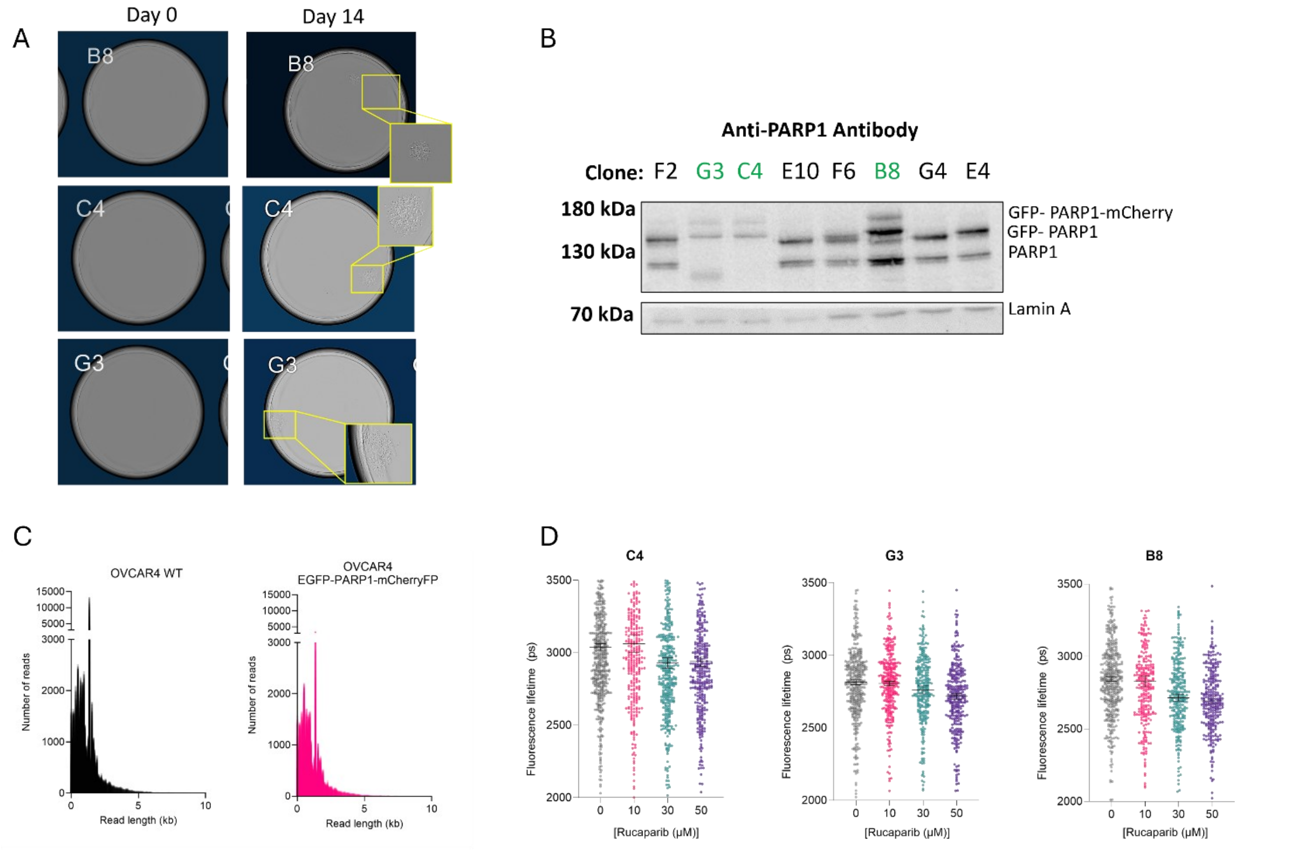

A: Representative images of three clonal populations of OVCAR4 EGFP-PARP1-mCherryFP cells when seeded at 0.5 cells/well (left) and after 14 days of growth (right), colonies highlighted in inset images. Images acquired with whole-well scans on the Incucyte S3. **B**: Eight clonal populations of OVCAR4 EGFP-PARP1-mCherryFP positive cells were isolated, western blots were performed to assess which clones had dual-labelled PARP1 alleles. Clones highlighted in green (G3, E4 and B8) showed dual labelled EGFP-PARP1-mCherryFP. **C**: Read length distributions for OVCAR4 WT (left) and OVCAR4 EGFP-PARP1-mCherryFP cells (right) from Oxford Nanopore direct long-read RNA sequencing on total RNA. **D**: Single-cell weighted mean fluorescence lifetime values from three clonal populations (C4, G3 and B8) of OVCAR4 EGFP-PARP1-mCherryFP cells exposed to 0-50 μM rucaparib for 1 h before FLIM acquisition. Error bars represent SEM, n=3 biological replicates.

Figure S2. Genomic cleavage detection assay for *BRCA1* mutant, related to Figure 3.

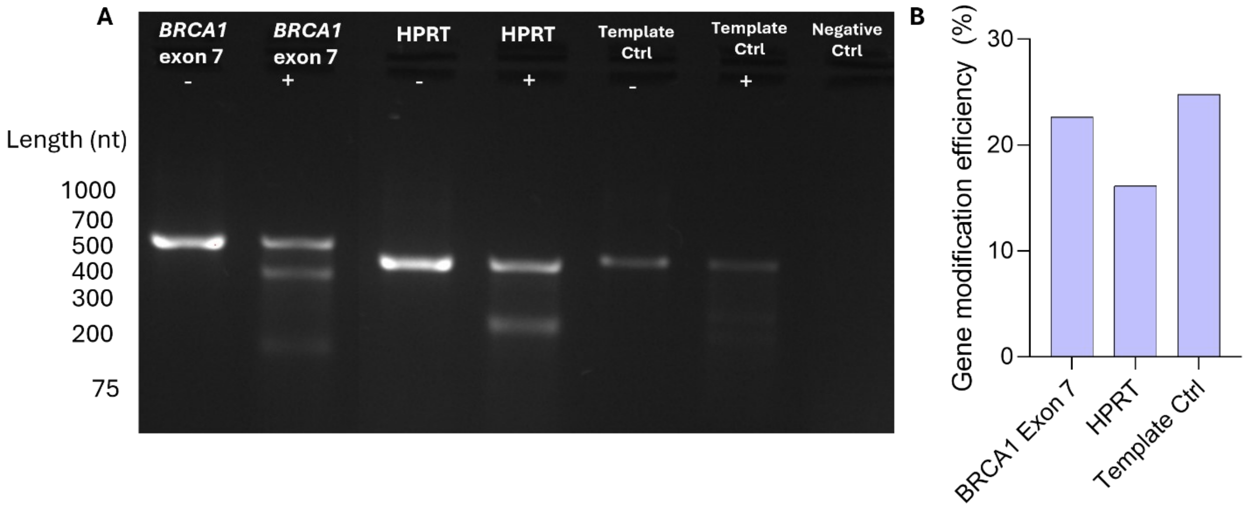

A: gel electrophoresis showing PCR amplicons from genomic cleavage detection (GCD) assay. Samples were analysed with (+) or without (-) T7 endonuclease I treatment. BRCA1 exon 7 shows cleavage products indicating successful CRISPR-Cas9-mediated targeting. HPRT served as a positive control for the assay, Template Ctrl represents the internal control provided in the GCD kit, and Negative Ctrl contains no DNA template. DNA ladder indicates fragment sizes in nucleotides (nt). **B**: Quantification of gene modification efficiency (%) calculated from band intensities in panel A. BRCA1 exon 7 showed 23% modification efficiency, comparable to the HPRT positive control (16%) and Template Ctrl (25%).

Figure S3. Cisplatin cross-resistance induced in PARPi treated cells, related to Figure 4.

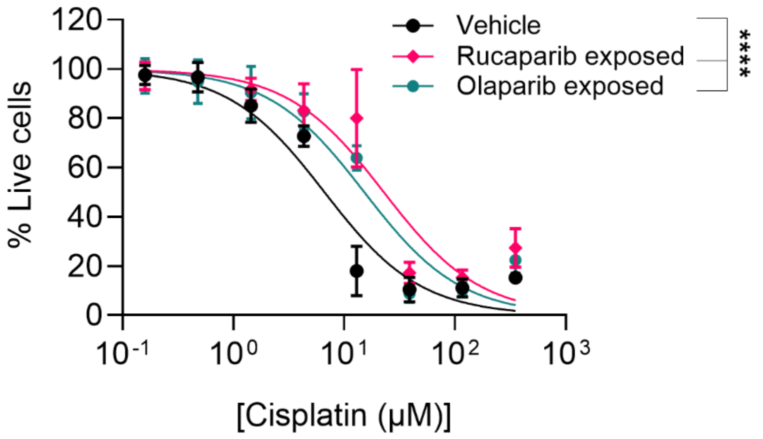

Viability assays were performed on the OVCAR4 EGFP-PARP1-mCherryFP cells following 9 weeks of continuous vehicle, rucaparib or olaparib exposure in vitro. Cells were treated with cisplatin for 72 h. Percentage of live cells are normalised to vehicle treated controls. Error bars represent SEM, n=3 biological replicates. T-tests were performed to compared IC50 values.

Figure S4. Segmentation of individual cells from rucaparib fluorescence, related to Figure 4.

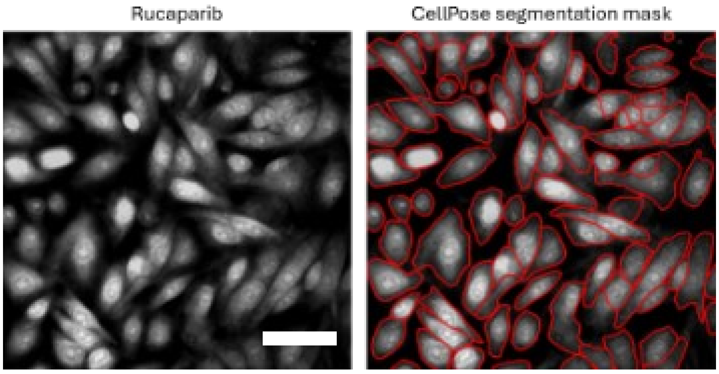

Individual cells were segmented from the rucaparib intensity images using CellPose 'cyto 3' model with default settings. Each cell in the segmentation mask was subsequently converted to an ROI within which the rucaparib intensity was quantified, giving a per-cell measurement. Scale bar = 25 µm.

Figure S5. Olaparib resistant tumours show no MDR1 upregulation by IHC, related to Figure 4.

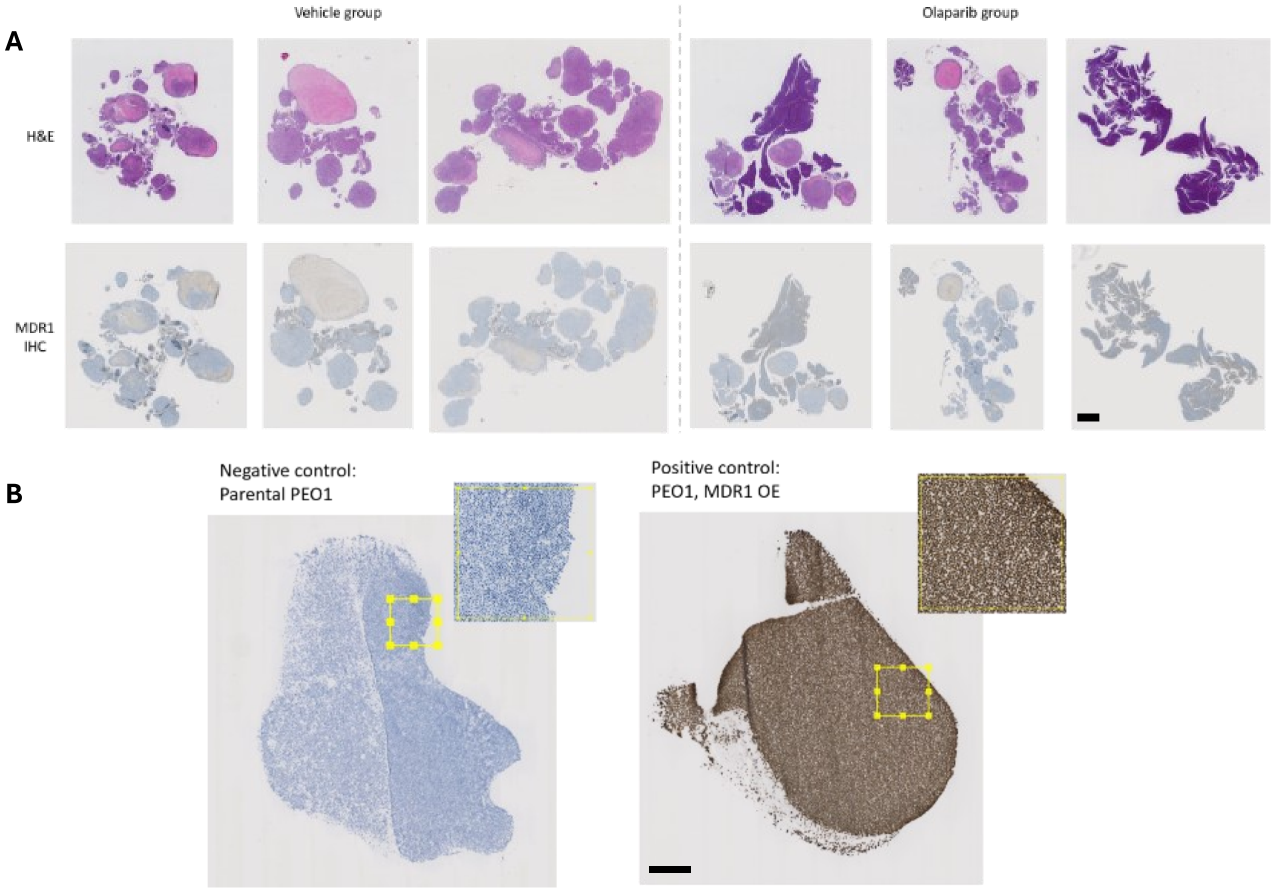

A: Representative H&E (top) and MDR1 IHC (bottom) images from omental tumour tissue harvested from vehicle treated (left) or olaparib treated (right) mice. n=3 biological replicates. **B:** Negative control for MDR1 expression cytospin of parental PEO1 cells (left) and PEO1 MDR1 overexpressing cells (right) as the positive control.
Scale bar= 2 mm.

Table S1. Oligonucleotides used in the paper, related to Figure 1,3,4,5.

Oligonucleotides		
TaqMan GAPDH Probe	Thermo Fisher Scientific	Hs02786624_g1
TaqMan PARG Probe	Thermo Fisher Scientific	Hs00608254_m1
TaqMan TP53BP1 Probe	Thermo Fisher Scientific	Hs00996827_m1
TaqMan ABCB1 Probe	Thermo Fisher Scientific	Hs00184500_m1
gRNA forward primer CACCGTCAATTTTAAGACCTCCCTG	Eurofins Scientific	N/A
gRNA reverse primer CAGTTAAAATTCTGGAGGGACCAAA	Eurofins Scientific	N/A
mCherry forward primer ATGGTGAGCAAGGGCGAGGAGG	Eurofins Scientific	N/A
mCherry reverse primer CGGCTACCTCTCCCAAttaccacagCTACTTGTACAGCTCGTCCATGCC	Eurofins Scientific	N/A
mCherry_arm5 forward primer GTGCCACCTGGGCCGGCCATTTAAATggcAGACAAGGATTAGAGGCTG	Eurofins Scientific	N/A
mCherry_arm5 reverse primer GCTCCTCGCCCTTGCTCACcatGGAGGTCTTAAAATTGAATTTCAGTTTCAGCAG	Eurofins Scientific	N/A
BRCA1 exon 7, forward primer CTTCCCAAAGCTGCCTACCA	Thermo Fisher Scientific	N/A
BRCA1 exon 7, reverse primer TCAAATCACACATATCCCACACA	Thermo Fisher Scientific	N/A
TrueGuide™ Synthetic sgRNA BRCA1 exon 7 (CRISPR813233_SGM) GAACTCTGAGGACAAAGCAG	Thermo Fisher Scientific	Cat #A35533
TrueGuide™ sgRNA Positive Control, HPRT1 (human)	Thermo Fisher Scientific	Cat # A35524
TrueGuide™ sgRNA Negative Control, non-targeting 1	Thermo Fisher Scientific	Cat # A35526
